# Transparent Wood Fabrication and Applications: A Review

**DOI:** 10.3390/molecules30071506

**Published:** 2025-03-28

**Authors:** Le Van Hai, Narayanan Srikanth, Tin Diep Trung Le, Seung Hyeon Park, Tae Hyun Kim

**Affiliations:** 1 Department of Materials Science and Chemical Engineering, Hanyang University, Ansan 15588, Gyeonggi-do, Republic of Korea; levanhai121978@gmail.com (L.V.H.); trungtinlediep@gmail.com (T.D.T.L.); rhcwlqdkemf@hanyang.ac.kr (S.H.P.); 2 Department of Chemical Engineering, University of New Brunswick, Fredericton, NB E3B 5A3, Canada; srikanth.naraayanan@gmail.com; 3 Major in Advanced Materials and Semiconductor Engineering, School of Semiconductor Convergence Engineering, Hanyang University, Ansan 15588, Gyeonggi-do, Republic of Korea

**Keywords:** bio-based materials, polymer resin, cellulose nanofiber, lignocellulose, delignification

## Abstract

Wood cellulose is an abundant bio-based resource with diverse applications in construction, cosmetics, packaging, and the pulp and paper industries. Transparent wood (TW) is a novel, high-quality wood material with several advantages over traditional transparent materials (e.g., glass and plastic). These benefits include renewability, UV shielding, lightweight properties, low thermal expansion, reduced glare, and improved mechanical strength. TW has significant potential for various applications, including transparent roofs, windows, home lighting structures, electronic devices, home decoration, solar cells, packaging, smart packaging materials, and other high-value-added products. The mechanical properties of TW, such as tensile strength and optical transmittance, are typically up to 500 MPa (Young’s modulus of 50 GPa) and 10–90%, respectively. Fabrication methods, wood types, and processing conditions significantly influence the mechanical and optical properties of TW. In addition, recent research has highlighted the feasibility of TW and large-scale production, making it an emerging research topic for future exploration. This review attempted to provide recent and updated manufacturing methods of TW as well as current and future applications. In particular, the effects of structural modification through various chemical pretreatment methods and impregnation methods using various polymers on the properties of TW biocomposites were also reviewed.

## 1. Introduction

Wood has long been a fundamental material widely used in various industries, such as pulp and paper, construction, flooring, furniture, and fuel. As the most abundant organic raw material on the planet, wood has played an essential role in human life for thousands of years. Recently, the commercialization of high-value-added wood for various uses other than traditional applications has attracted increasing research attention. In particular, wood has a low carbon footprint, is environmentally friendly and is renewable.

Among its various emerging applications, transparent wood (TW) has gained significant attention for its potential use in high-value-added products, such as smart windows, rooftops, decorative materials, solar cells, insulation, lighting management, and UV shielding [1,2,3,4,5,6,7,8,9,10]. Fink first introduced TW prepared by impregnating (infiltrating) different types of polymers into wood structures [11]. Previous studies [9,10,11,12,13,14] have highlighted the numerous advantages and potential future applications of TW. TW intended for commercial and industrial use must be environmentally friendly and safe for the human body, ensuring the absence of toxic or harmful chemicals.

Transparent wood fabrication begins with the chemical pretreatment of lignocellulosic biomass, involving either of the following processes: (1) complete lignin removal [11] or (2) chemical modification of lignin with partial delignification while retaining a controlled amount of lignin [8,15,16,17,18]. To produce a high-quality transparent wood material, the delignified wood is impregnated with a polymer with a refractive index (RI) closely matching that of the wood matrix [11]. Subsequently, the RI difference between the cell wall and lumen is reduced, resulting in less light scattering and higher transparency. In recent studies, various polymers, including epoxy, prepolymerized methyl methacrylate (PMMA), polyvinyl pyrrolidone (PVP), polyethylene glycol/methyl methacrylate (PEG/MMA), polyvinyl alcohol (PVA), polyurethane (PU), epoxy vitrimers, TEMPO-treated cellulose nanofiber (CNF), and chitosan (CTS) polymer, have been used to make TW [14,15,17,19,20,21,22]. However, in recent years, various environmental issues have placed many restrictions on using conventional petrochemical-based resin materials (e.g., epoxy or PMMA). Consequently, bio-based polymers, such as limonene acrylate monomer, CNF, and CTS [14,23], have been increasingly used to produce eco-friendly TW. In addition, self-densified and compacted TW has demonstrated enhanced mechanical properties [24,25].

Light transmittance is the most critical property of TW, typically influenced by wood thickness, delignification process, light transmittance of the polymer, and difference in light transmittance between the polymer and wood. Previous studies have reported transmittance values of TW varying from 10% to 90% [1,2,5,10,17,21]. Several factors, including wood species and type, delignification process, bleaching conditions, thickness, tensile strength, expansion rate, light transmittance, and polymer properties, influence the performance of TW [2,5,17]. Owing to the limitations of conventional glass, such as brittleness and low thermal insulation, developing a method for producing commercially viable TW with enhanced tensile strength and less brittleness is crucial. Studies have demonstrated that the mechanical properties of TW could be substantially improved. For example, reinforcing with epoxy resin significantly improved its longitudinal tensile strength from 42.7 MPa to 45.4 MPa and from 4.5 MPa to 23.4 MPa in the radial direction [21]. The improvement in the tensile strength of TW was reported to be approximately 106% and 520% in the longitudinal and radial directions, respectively, corresponding to 10 MPa to 200 MPa or higher [1,5,13]. Furthermore, multilayered TW has been developed to achieve enhanced mechanical properties in all directions [9,26,27]. By contrast, some studies have reported a reduction in mechanical strength, with reductions from approximately 220 MPa to approximately 150 MPa [2,19] or from 60 MP to 30 MPa [28,29].

For the industrial application of TW, understanding the influence of raw materials and fabrication processes on its mechanical properties is crucial. This review explores the various types of TW, their production methods, and the impact of polymer impregnation and manufacturing processes on the mechanical and optical properties of TW. Furthermore, it explores advancements in nanostructured materials, high thermal insulation, and thermal conductivity. This review examines various factors affecting the transmittance of TW. Furthermore, it explores factors affecting transparency, such as wood types, polymers used, material thickness, and manufacturing processes.

## 2. Review of the Fabrication Process of TW

**Figure 1:**
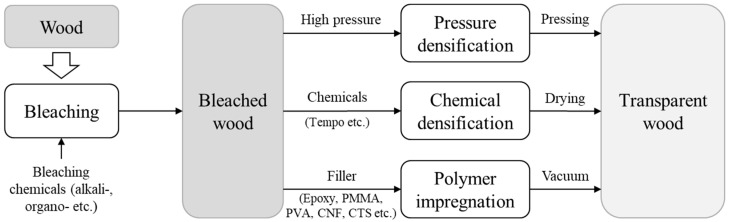
General fabrication methods of TW.

Currently, softwood and hardwood species are used in composites TW with various polymers, including PMMA, PVA, PVA-lignin nanoparticles, PVA-chitin nanofibers, chitin nanocrystals, chitin-chitosan [8], CNF, CTS, PU, and PEG/MMA. Various bleaching agents (catalysts) are used, such as NaClO_2_, H_2_O_2_, NaOH, acetic acid, sodium acetate, ethylenediaminetetraacetic acid (EDTA), and other combinations are used to delignify wood-based raw materials. Figure 1 shows the general process for producing TW.

### 2.1. Bleaching and Delignification

**Figure 2:**
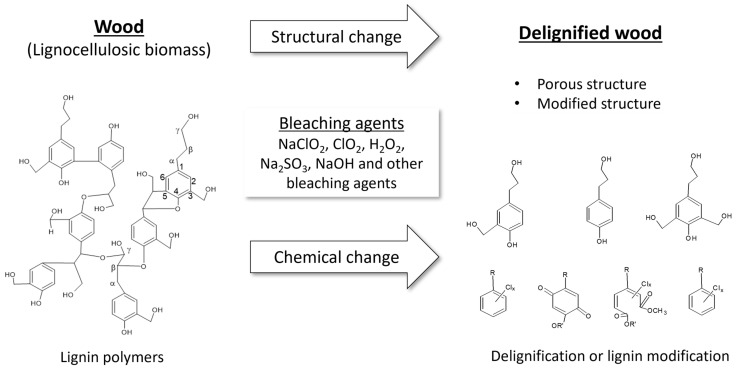
Delignification, lignin modification, and the development of porous wood structure through bleaching [16,32,33,34].

Delignification is a crucial initial step in producing TW, although complete lignin removal is not always necessary [15,16,18,30]. Several studies [15,16,17] have indicated that even partial lignin removal from wood sources can result in TW. Various bleaching and delignification methods are used in the early stages of TW production. Wood delignification involves using alkalis, organic acids, NaClO_2_, H_2_O_2_, and enzymes. Bleaching conditions influence the transmittance and mechanical properties of TW. Wood type, wood thickness, reaction time, and chemical dosage also affect the bleaching process. A common delignification method involves oxidative bleaching. This process involves treating dried wood by immersing it in a sodium chlorite (NaClO_2_) bleaching solution with acetate buffer, maintained at a pH of 4.5 and temperature of 70–80 °C. As mentioned in the Introduction, bleaching using NaClO_2_ is the preferred method for producing TW. Other studies used a combination of H_2_O_2_ in the delignification process [21,28,31]. Wood can be bleached using different methods, bleaching agents, temperatures, and durations. Bleaching removes lignin content, alters its color, and transforms brown wood into a white one. Lignin modification retains lignin while decolorizing the wood. The bleaching process breaks down the bonds within the complex lignin structure. The most easily cleaved lignin bonds include α-O-5, β-O-5, and γ-O-5. The mechanisms involved in the bleaching process have been widely studied in the pulp and paper industry. Details on wood and pulp bleaching are discussed in several studies [16,32,33,34]. Highly delignified and bleached wood turns white and exhibits a highly porous structure, facilitating efficient polymer infiltration. However, lignin modification primarily whitens or decolorizes wood. This approach has gained significant interest in the large-scale and efficient production of TW, as demonstrated by [35]. Their study highlighted the potential of lignin modification in producing TW and its viability for large-scale production. Xia et al. applied NaOH and H_2_O_2_ to wood samples, followed by UV illumination, until the samples turned completely white [35]. Subsequently, various TW products were prepared through vacuum infiltration using epoxy (300/21 epoxy resin, Aeromarine Products Inc., San Diego, CA, USA). TW prepared through lignin modification has the potential for various applications and large-scale production. Figure 2 illustrates the lignin modification and delignification processes, along with their mechanisms.

A washing step is performed after bleaching to remove unreacted chemicals, extracts, lignin, and impurities. During this process, the pH is neutralized, and a light vacuum may be applied to the pretreated wood to remove residual chemicals and volatile components. Subsequently, the treated wood undergoes a solvent-exchange process, where it is immersed in an ethanol and acetone solution. Depending on the purity, the amount of solvent required for the exchange process typically ranges from two to three times the volume of wood. After undergoing these processes, the wood material is ready for TW production. However, solvent exchange is not always necessary. TW can be produced by directly infiltrating the polymer into the dried, bleached wood without a solvent-exchange process. Table 1 lists the different delignification and bleaching agents used for TW fabrication.

### 2.2. Impregnation of Bleached Wood with Various Polymers

#### 2.2.1. Previous Approaches of Impregnation Process

Previous studies have identified resin (filler) penetration as the preferred and most effective method for producing TW using materials obtained after the initial delignification process [2,38,39]. Delignified and bleached wood materials typically have empty spaces from which components such as lignin and hemicellulose are removed, resulting in a porous structure. To achieve transparency, these pores are filled with a material with a refractive index close to or equal to that of wood. In some cases, the selected polymers also provide UV-shielding properties. According to Fink [11], wood generally has a refractive index of approximately 1.53. Commonly used polymers have refractive indices ranging from 1.50 to 1.53, making polymer resin suitable for TW fabrication. The delignified porous wood material is filled with polymer through impregnation or penetration. Various types of polymers, such as PMMA, PVP, PU, epoxy resin, quantum dots, PVA-lignin, PVA-lignin nanoparticles, PVA-chitin nanocrystals, chitin crystal, PEG/MMA, ATO/MMA, CNF, and CTS, have been used for this purpose [1,2,6,7,8,11,15,19,20,21]. Matching the refractive index (RI) of polymer for TW production is one of the important factors. However, it is difficult to match the various RIs depending on the polymer structure to produce TW. Polymers used in TW, including PMMA, epoxy resin, and PVA, may contain SiO_2_, TiO_2_, and lignin to modulate the RI of composite materials. These nanoparticles may play a role in controlling the RIs of polymers and wood. However, further study is needed to evaluate the properties of TW composite materials when infiltrated into TW.

Polymer impregnation typically takes 30 min to several hours and involves repeated cycles of impregnation, release, and penetration. However, the low specific gravity of wood during impregnation makes effective polymer infiltration into the porous wood challenging. Alternative methods, such as compression or self-densification of oxidized wood using TEMPO and other solvents, have been explored to improve polymer penetration. For instance, Zu et al. [24] produced TW using a high-pressure compression method on bleached wood.

#### 2.2.2. Pressurization of Delignified Wood

Li et al. produced TW by densifying wood after TEMPO oxidation [25]. Samanta et al. have prepared TW biocomposites for smart window applications [40]. These TW composites were prepared using thiol and ene monomers containing chromic components and a mixture of thermo- and photo-responsive chromophores. To produce TW through pressing, the lignin content of the wood materials must first be removed. Different bleaching chemical agents are used for delignification, such as NaClO_2_, NaClO, and H_2_O_2_. After lignin is removed from the natural wood, the resulting bleached wood is subjected to pressurize between microporous filtering films for 1 h to 3 h and subsequently dried.

#### 2.2.3. Polymer Infiltration

Wood materials can be processed with or without delignification and bleaching to produce TW. When bleaching or delignification is performed, several chemical agents (NaOH, Na_2_SO_3_, NaClO_2_, NaClO, and H_2_O_2_) are used to remove a portion or the entire lignin content from the wood substrate, resulting in different types of TW [2,17,18,23]. Depending on the lignin content, TW can be categorized into lignin-retaining, partially bleached, and fully bleached TW. To prepare TW, pretreated wood (processed through solvent exchange using ethanol or acetone) or wood bleached using bleaching agents (subjected to solvent exchange using ethanol or acetone) is infiltrated with various polymers, such as epoxy resin, PMMA, quantum dots, PVA, PLA, chitosan, and cellulose nanofibers through a vacuum process [1,2,16,21,31]. This renders the wood transparent, with varying levels of transmittance and haziness.

The infiltrated components used for fabricating TW for smart windows consisted of pentaerythritol tetrakis (3-mercaptopropionate) (PETMP), 1,3,5-triazine-2,4,6(1*H*,3*H*,5*H*)-trione (TATATO), and the UV initiator 1-hydroxy-cyclohexyl phenyl ketone (0.5%). In addition, Hai et al. have developed bio-based TW for packaging and wood straws by infiltrating wood with dissolved chitosan and TEMPO-nanocellulose [14]. A study by Qiu et al. explored the transition from high-haze wood to TW using phase-change materials [41]. They used various copolymers, including styrene (St), butyl acrylate (BA), and 1-octadecene (ODE), for the infiltration. At low temperatures (e.g., 5 °C), the wood exhibited high haze and low transparency. However, increasing the temperature to 25 °C or 50 °C for 2 min rendered the wood transparent. To prepare thermo-responsive flexible TW, the authors infiltrated wood with a combination of (St/BA/ODE) St and BA (1:1 ratio) containing 0.3% divinylbenzene (DVB, 55%) as the cross-linker and 0.2% 2,2′-azobis-(2-methulpropionitrile) (AIBN) as the initiator [41]. The mixture was precopolymerized at 85 °C for 35 min. By adding 5% 1-octadecene (ODE 90%) to the St/BA mixture, a thermo-responsive flexible opaque and TW composited was prepared. Table 2 presents the various methods used for TW fabrication and their applications.

#### 2.2.4. Self-Densified TW

Self-densified TW is a recent method that involves bleaching using sodium chlorite (NaClO_2_) and an acetate buffer (pH 4.6) at 80 °C for 12 h. The natural wood is transformed into white wood, washed, and immersed in a 0.1 m sodium phosphate (PBS) solvent. Subsequently, the wood is treated using the TEMPO oxidation method. Following this, the wood is dried at ambient temperature to produce self-densified TW [25].

## 3. Physical Properties of TW

### 3.1. Optical Transmittance and Haziness

Transmittance is the most crucial property of TW. A higher transmittance allows more light to pass through, improving visibility. Light transmittance in TW is primarily influenced by wood thickness, bleaching conditions, type of impregnated polymers, resins, and wood type.

High light transmittance and sufficient wood thickness are crucial for the successful industrial or commercial application of TW. However, while thicker wood is suitable for construction, it tends to have lower transmittance. In general, a thicker TW exhibits higher blurriness and lower light transmittance, making it challenging to achieve clear visibility.

TW with high transmittance or translucency is preferred for applications such as solar cells, light management, and structural elements. However, products requiring high transparency demand minimal haze. Therefore, applications in buildings and residential areas require a balance between transmittance and visibility. Yaddanapudi et al. reported that TW made from beech and PMMA exhibited transmittance ranging from 10 to 70%, depending on the wood thickness (0.1 mm to 0.7 mm) [2]. Balsa wood has been widely used for manufacturing TW, with transmittance ranging from 10 to 90%, depending on the thickness [1,6,10,15]. Several studies have reported methods for preparing TWs of various thicknesses. For example, Fu et al. reported TWs with a thickness of 3.5 mm and a transmittance of about 70–90% depending on the infiltrated polymer component [6], and Mi et al. reported various types of TWs for aesthetic wood applications with a thickness of 2 mm and a transmittance of about 80% [10]. Hai et al. recently reported various types of TWs with very thin layers for various applications with a transmittance of about 70–80% [14]. In addition, Hai et al. combined PVA and lignin nanoparticles to produce TWs with a thickness of 1–2 mm for UV-blocking window applications [8,9]. High turbidity and high transmittance are prioritized based on the TW application. For example, high-transmittance and high-haze products are suitable for applications such as solar cells, outdoor displays, and home lighting management.

In a recent study, Jia et al. [29] developed TW with a transmittance of approximately 90% and haze of 10%, demonstrating clear visibility and high potential as a building material.

### 3.2. UV-Shielding Properties

Another notable advantage of TW is its UV-shielding properties. Numerous studies on TW and glass have shown that, unlike glass with little to no UV-shielding properties, TW exhibits excellent UV-shielding capabilities. TW with different types of filler materials holds significant potential for UV-shielding applications. UV rays from the sun are known to have various negative effects on humans, such as skin burns and cancer. Therefore, TW with high UV-shielding properties offers a significant advantage for commercial success [7,8,10,13]. TW produced by infiltrating PMMA/antimony-doped tin oxide (ATO), PMMA, NCF, and CTS solutions has shown significant UV-shielding capability, with transmittance demonstrating high UV protection properties (~80%) in the 200–400 nm range. Furthermore, recent developments have explored partial delignification and reinforcing lignin nanoparticles to enhance UV shielding [8,14]. In one study, bleached wood was infiltrated with a composite of PVA and lignin nanoparticles to create UV-shielding TW. In addition, a simpler approach involving delignification using NaOH without bleaching was demonstrated to produce TW with UV-shielding properties [8,14]. Therefore, UV-shielding properties offer significant advantages for window applications.

### 3.3. Density, Modulus, Strength, and Toughness

Wood is a natural material with several unique properties, such as lightweight and high tensile strength, Young’s modulus, and toughness. These properties vary depending on the wood species, growth conditions, and whether it is earlywood or latewood. In general, the density of wood is approximately 0.65 g/cm^3^. Studies have reported that the density of latewood and earlywood in Douglas fir ranges from 300 kg/m^3^ to 800 kg/m^3^ [10,14]. Another previous study reported Balsa wood with a density ranging from 112 kgm^−3^ to 170–200 kgm^−3^ [25,40]. TW is a suitable alternative for building and construction applications owing to its superior properties compared with other materials, such as natural wood, steel, and glass. Therefore, mechanical properties, such as tensile strength, Young’s modulus, toughness, and density, should be considered. The literature reviews have confirmed that the mechanical properties of TW are superior to those of natural wood [5,10,14,38]. Wang et al. reported that natural wood had a tensile strength of approximately 42.8 MPa and a modulus of 5.7 GPa [38]. By contrast, TW exhibited an average tensile strength and modulus of 52.3 MPa and 2.4 GPa, respectively, with elongation at break increasing from 1% to approximately 5% compared with untreated wood. Mi et al. reported that TW exhibited significantly enhanced tensile strength and toughness compared with natural wood [10]. The tensile strengths of natural wood in the radial and longitudinal directions were less than 10 MPa and approximately 40 MPa, respectively, whereas those of TW exceeded 20 MPa and 95 MPa, respectively. In addition, Mi et al. showed that the toughness of TW increased fivefold in the radial and longitudinal directions [10].

### 3.4. Thermal Conductivity

Heat transfer is a key factor in construction and building applications. Modern architecture increasingly features high-rise or large-scale buildings with open designs and extensive glass windows to allow natural light into the buildings. Glass has traditionally been the preferred material for windows. However, glass windows have several disadvantages, such as high thermal conductivity, heavyweight, and brittleness. TW is an emerging material with significant advantages over glass, such as lightweight properties, ductility, UV shielding, and low thermal conductivity. Wood is known to be a low-thermal-conductivity material, making TW a promising alternative for reducing thermal conductivity. Several studies have explored the thermal conductivity and UV-shielding properties of TW [4,5,10,13]. These studies have reported that TW exhibited 3–4 times lesser thermal conductivity (0.32–0.15 W m^−1^ K^−1^) than glass (1.0 W m^−1^ K^−1^). Thus, large-scale production of TW is expected to play a significant role in future construction applications.

## 4. Factors Affecting Optical and Mechanical Properties of TW

The mechanical characteristics of TW are one of its critical properties, and they vary based on their intended use. As previously discussed, TW is used in home structures, smart houses, packaging, and glass substitutes. The mechanical properties of TW used for these applications are primarily influenced by the wood type, processing conditions, and type of impregnating polymer used. To function as a substitute for fragile glass, TW must have the mechanical strength required for its use as a building material. Zu et al. demonstrated that simple bleached and compressed TW exhibited superior mechanical properties compared with wood impregnated with PMMA, epoxy resin, PVA, and NCF [24]. However, TW produced through compression alone had the disadvantage of limited thickness increase. In a recent study, Li et al. reported that TW produced through TEMPO oxidation and high-pressure compression exhibited high tensile strength and Young’s modulus, reaching approximately 500 MPa and 50 GPa, respectively, despite being a relatively thin material [25].

TW impregnated with PVA and PMMA exhibited extremely poor mechanical properties. This can be attributed to various factors, such as wood type, bleaching conditions, and other processing factors. According to Jungstedt et al. [42], birch exhibited an initial longitudinal tensile strength of approximately 167 MPa, while materials made by infiltrating PMMA under different conditions achieved tensile strengths of 193 and 263 MPa. Yu et al. [39] reported that natural wood had a strength of 55.1 MPa, increasing to 60.1 MPa following PMMA impregnation. Qui et al. [41] noted that native Balsa wood had a strength of approximately 20 MPa, whereas polymer-impregnated materials exhibited a tensile strength of approximately 10–20 MPa, depending on the resin type and penetration conditions. Research indicates that the type of wood used in manufacturing TW significantly influences its mechanical properties and tensile strength development.

Table 3 lists the mechanical properties of various wood types and TW in the longitudinal (_//_) and radial (_⊥_) directions. In addition, it summarized the effects of bleaching agents, bleaching conditions, wood thickness, and wood type on transmittance.

## 5. Potential Applications of TW

### 5.1. TW for Building and House Structure

Previous studies [2,5,11,16] have highlighted the potential applications of TW, including housing structures, smart houses, walls, and rooftops. For example, several studies [1,39] have shown that CSxWO3/PMMA composites exhibited excellent insulation properties, making them ideal for use as window materials. Li et al. developed TW of various thicknesses, ranging from 20 mm to 50 mm [5]. The 20 mm thick TW impregnated with epoxy resin exhibited a transmittance of up to 40%, making it suitable for use in wall structures. In addition, Li et al. reported that TW made by impregnating epoxy resin exhibited significantly enhanced transmittance compared with TW infiltrated with PMMA [5]. Therefore, to develop advanced TW for housing structures, reducing the turbidity of TW and further exploring technologies related to the use of various woods and fabrication processes are crucial.

### 5.2. Light Management, House Decoration, Solar Cells and Electric Devices

TW has potential applications in buildings, lighting management, and home decorations [3,4,10,13,39]. It can be used in rooftops, lighting management, and decoration. Yu et al. reported that TW resulted in enhanced heat-shielding properties compared to glass [39]. Model houses with CsxWO3/MMA TW windows exhibited better insulation than existing houses using ITO glass windows, resulting in nearly double the indoor temperature. According to Li et al. [1], TW exhibited minimal glare, and Lang et al. [43] noted that TW infiltrated with PMMA and coated with PETDOT:PSS demonstrated significant potential as a glass substitute for windows. In addition, Mi et al. [10] noted that TW could improve lighting and insulation and create pleasant interior lighting. These properties make TW a promising material for smart houses, buildings, and windows.

In addition, TW can be used in other industries, such as electronics, sensors, and solar cells. Recent studies [5,19,21,38] have demonstrated its potential in lighting management, solar cell materials, thermal energy storage systems, and energy-saving applications. Wang et al. [38] revealed that TW and transparent-wood-based fibers exhibit extremely low thermal conductivity of approximately 0.2 W/mK. By contrast, ITO glass has a thermal conductivity of approximately 1.0 W/mK, approximately five times higher than that of TW. This makes TW a viable energy-saving material for buildings. Montanari et al. [19] produced TW by immersing delignified wood in a PEG/MMA (70/30 *w*/*w*) polymer solution three times. The resulting TW exhibited excellent thermal energy storage and energy-saving potential. Li et al. [5] suggested combining solar cells and wooden building materials as an energy-saving solution. Other studies [5,21] explored TW for solar cell applications, reporting conversion efficiencies ranging from 14.4% to 16.8%. Another study used TW to fabricate perovskite solar cells, consisting of a TW/ITO layer/TiO_2_/perovskite/spire-OMeTAD/Au layer. The perovskite solar cells exhibited a current density of 21.9 mA·cm^−2^, voltage of 1.09 V, and charge rate of 70.2%. These findings indicate that TW is a promising eco-friendly housing material.

### 5.3. TW as Green Bio-Based Packaging Materials

**Figure 3:**
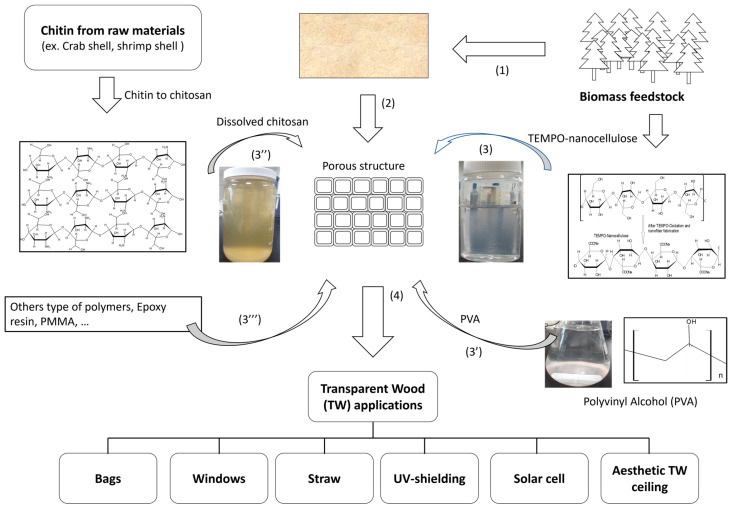
Fabrication process and applications of TW: (1) from wood-to-veneer and TEMPO-treated nanocellulose; (2) bleached wood; (3) TEMPO-treated nanocellulose; (3′) PVA; (3″) chitosan; (3‴) other types of polymers; (4) TW applications; TW bag, window, straw, solar cells, and aesthetic wood ceiling [14,16,28,35,39,41,43,45].

Bio-based TW, fabricated by impregnating CNF and CTS suspensions into bleached porous wood, has demonstrated excellent antioxidant and UV-shielding properties, improved mechanical properties, and enhanced transmittance. Hai et al., 2021 produced bio-based TW in significantly larger sizes (200 mm × 100 mm × 0.1 mm) compared with those in previously published works and used two types of TW for packaging applications [14]. This highly flexible material can be used for transparent straws, transparent bags, and medical window packaging. However, despite the many advantages of bio-based materials, such as cellulose nanofibers and chitosan solutions, hydrophilicity remains a significant challenge. Further efforts are necessary to address this limitation and improve the thickness of bio-based TW. Figure 3 illustrates the fabrication process and TW applications. In addition, Zhang et al. recently also developed biobased transparent wood for food packaging [44]. The authors demonstrated that food packaging has good UV-shielding functionality, antioxidation, and so on.

### 5.4. Patents on TW

TW is a promising material for developing green products, potentially replacing traditional window glass, structural components in buildings, and decorations and improving sunlight management. TW development has rapidly progressed in recent years. However, the emerging trend of bio-based materials and their feasibility for real-world applications has led to the filing of several patents worldwide. Advanced TW offers excellent mechanical properties, ease of fabrication, low thermal expansion, and minimal environmental impact. Consequently, research groups, companies, and institutions have increasingly pursued patents related to TW. In 2017, Cellutech filed a patent for a method of preparing TW [46]. Cellutech listed a range of polymers for TW fabrication, such as poly(hexafluoropropylene oxide), hydroxypropyl cellulose, poly(tetrafluoroethylene-co-hexafluoropropylene), poly(pentadecafluoro octyl acrylate), and poly (tetrafluoro-3-(heptafluoropropoxy) propyl acrylate), among others. In 2017, the University of Maryland secured a patent for TW with a high transmittance of 92% [47]. The patent lists various polymers for TW fabrication, including polyester fiberglass, polyurethane polymers, vulcanized rubber, bakelite, duroplast, urea-formaldehyde, melamine resin, diallyl phthalate (DAP), polyimides and bismaleimides, and cyanate esters or poycyanurates, among others [46,47]. In addition, they patented a process and various polymers for creating bio-based TW. With TW being an emerging field with significant potential for industrial applications, research since 2016 has led to several patents from companies, universities, and institutions [46]. As the development of TW continues, more patents and industrial applications are expected to emerge in the future.

### 5.5. Future Trends and Challenges

TW has gained significant research attention in recent years owing to its potential applications and unique properties. However, several challenges remain, such as high hydrophilicity, permeability, production scalability, and chemical consumption. To address these challenges, researchers have proposed various solutions, including hydrophobic coatings with materials such as silicates and quantum dots, improving the thickness of bio-based TW through layer-by-layer deposition.

Currently, three primary approaches are employed for TW production: penetration, compression, and self-densification of TEMPO-oxidized bleached wood. Although conventional chemical polymers, such as PMMA, epoxy resin, and PVA, were initially used for impregnation, recent research has expanded the selection to include various materials. Combinations of different polymers are also being explored, although conventional fillers, such as PMMA and epoxy resin, remain toxic and harmful to human health and the environment. By contrast, bio-based materials, such as PVA, CNF, and CTS, are nontoxic but exhibit poor physical properties, including water absorption and hydrophilicity. Nevertheless, bio-based materials, such as PLA, CNF, and CTS, are preferred owing to their minimal environmental impact and lack of human hazards. In addition, CNF are considered as a potential material to infiltrate porous wood to produce TW, but the high energy cost for production of CNF to be reduced. The high energy consumption and waste generation and disposal costs due to the use of chemicals in CNF production remain issues that will be solved in the future. However, the high hydrophilicity of these materials necessitates a hydrophobic coating for the resulting TW to prevent moisture absorption and humidity damage caused by weather changes.

The size and thickness limitations of TW pose challenges for industrial and commercial applications. However, Xia et al. [35] successfully fabricated meter-scale TW with a thickness of 1 mm, marking a significant breakthrough in production scalability. Consequently, research focused on commercialization is expected to accelerate in the near future. Overcoming these challenges holds significant potential for future applications of bio-based TW, particularly in improving mechanical properties, thermal expansion, light management, and the customizing of thickness and filler content for improved flexibility and toughness. Ongoing research focuses on optimizing hydrophilicity and hydrophobicity through various physicochemical methods, which can further expand the potential of TW for various applications.

## 6. Conclusions

The potential applications of TW primarily include materials for residential building structures, smart houses, windows, solar cells, and packaging, with significant expansion anticipated in the future. High-density TW of consistent thickness is suitable for building, window, and home structural applications. However, it can also be converted or fabricated into a thin film, making it suitable for rolling, decorative, and packaging purposes. Currently, the primary methods for manufacturing TW include penetration, high-pressure compression, and self-densification.

The fabrication of TW using eco-friendly materials such as NCF, CTS, and other bio-based materials has great potential. However, various eco-friendly polymer materials and manufacturing methods for TWs with desirable mechanical and functional properties should be developed. In particular, the mechanical properties of TW, including tensile strength, Young’s modulus, and transmittance, are greatly influenced by wood species, polymers, and processing methods. As highlighted in this review, eco-friendly TW offers significant market potential and a wide range of applications.

This paper provides a review of the latest and updated manufacturing methods, properties, and applications of bio-based composite material (TW). In particular, the effects of structural modifications using various chemical reagents and combinations using various polymers on the properties of TW biocomposites were also examined.

## Figures and Tables

**Table 1:** Bleaching methods using various woods and bleaching agents for TW production.

Wood Species; Size (W:L:T)	Bleaching Agents and Delignification	References
Poplar veneer; 80:80:3 mm^3^	KOH (>98%) and DI water followed by NaClO (>98%) for 8 h at 120–130 °C. The amount of lignin content before and after bleaching is not indicated.	[36]
Basswood	Boiling with NaOH (2.5 mol L^−1^) and Na_2_SO_3_ (0.4 mol L^−1^) for 12 h. Second Step: H_2_O_2_, 2.5 mol L^−1^ for 12 h. In the first stage, lignin content = ~12–14%; second stage, the lignin content ≤ 3.0%.	[21]
Basswood	Soaking in NaOH (2.5 mol/L) and Na_2_SO_3_ (0.4 mol/L). Boiling for 12 h. Bleaching with H_2_O_2_, (2.5 mol/L). This resulted in 33%, 50%, and nearly 100% lignin removal.	[37]
Poplar (*Populus* sp.) and Balsa wood. Width (80–300 mm), Length from 25 to 300 mm and thickness from 1–10 mm	NaOH (10 wt%) and Na_2_SO_3_ (5 wt%) boiling for 2–4 h, followed by boiling in DI water. Further whitening using H_2_O_2_ (30 wt%) in boiling. The lignin content is ~2.8%.	[38]
Balsa (*Ochroma pyramidale*), alder (*Alnus glutinosa*), birch (*Betula pendula*), and beech (*Fagus sylvestris*); 0.7–3 mm thickness	Peracetic acid (PAA) and CH_3_COOOH. Treated at 80 °C using aqueous PAA solution (4 wt%) at a pH of 4. 8 (adjusted with NaOH), followed by washing with DI water and acetone. Lignin removal from 18.2% to 27.9% of untreated to 0.9 to 2.0% of treated biomass.	[23]
Basswood (*Tilia*); 20:20:0.42 mm^3^	2.0 wt% NaClO_2_ and 0.1 wt% acetic acid glacial, bleaching time for 30, 60, 90, 120, and 150 min. This resulted in lignin removal of 33, 38, 47, 51, and 64%, with treatment time of 30, 60, 90, 120, and 150 min, respectively.	[17]
Basswood (*Tilia*); 20:20:0.42 mm^3^	6.0 wt% H_2_O_2_, 1.0 wt% trisodium citrate 95% dihydrate, 1.0 wt% of NaOH, and 92 wt%. Bleaching time of 30, 60, 90, 120, and 150 min, and lignin content varied from 24.3, 19.5, 18.4, 16.6, 15.2 and 14.9 wt%.	[30]
Beechwood	NaClO_2_ (5.0 wt%) in acetate buffer solution at 95 °C for 12 h. The lignin content was not indicated.	[2]
Birch (*Betula alnoides*, *Betula*) and New Zealand pine (*Pinups radiata D. Don*); 20:20:0.5 mm^3^	NaClO_2_ (0.4–1.0%) at 70–90 °C for 45–135 min. The pH 4.6 was adjusted by adding CH_3_COOH.	[18]

**Table 2:** TW fabrication methods and applications.

Wood Species	Fabrication Method and Filler (Polymer)	Applications	Ref.
Basswood	15 wt% Polyvinylpyrrolidone (PVP) ethanol. Degassed under 200 Pa for ~10 min	Highly efficient broadband light management in solar cells	[37]
Poplar (*Populus* sp.) and Balsa wood	Impregnation using vacuum infiltration process with prepolymerized MMA solution (PMMA)	TW for Energy-saving building	[38]
Silver birch wood (*Betula pendula*)	Impregnation using vacuum infiltration with PEG/MMA (70/30 wt%) solution	Thermal energy storage and reversible optical transmittance	[19]
Balsa wood	Impregnation using vacuum infiltration with polyvinyl alcohol (PVA) solution	Thermally insulated TW for energy-efficient windows	[13]
Douglas fir, Bass, Balsa, and Pinewood	Infiltration into the delignified wood scaffold using epoxy resin, followed by solidification for 24 h	Aesthetic TW for energy-efficient buildings	[10]
Basswood	Impregnation with 2 epoxy resins (E-128 resin and D-630) at a mass ratio of 3:1	Thick translucent wood walls and indoor light performance	[5]
Balsa wood	Infiltration using poly-methyl methacrylate (PMMA) and laser deposition for conductive film layer using indium tin oxide (ITO).	TW substrate for perovskite solar cells	[4]
Beechwood	Impregnation using vacuum infiltration with poly methyl methacrylate (PMMA) for 1 h with three repetitions, followed by heat treatment in a box furnace at 85 °C for 12 h	Next-generation smart building materials	[2]
Beechwood	Impregnation using vacuum infiltration with CsxWO_3_/prepolymerized methyl methacrylate (MMA) mixed solution, followed by infiltration for 30 min with three repetitions	TW containing CsxWO_3_ nanoparticles for heat-shielding-window	[39]
Fir wood	Impregnation using vacuum infiltration with TEMPO-treated nanocellulose and chitosan solution	Food packaging materials, medical packaging materials, and straw	[14]
Balsa wood	Impregnation using vacuum infiltration with PVA, PVA-lignin nanoparticle	UV-shielding windows application	[8]

**Table 3:** Various mechanical properties of TW from different wood sources.

Name of Wood	Wood Sizes (mm)	Bleaching Agents	Polymer	Tensile Strength (MPa)	Young’s Modulus (GPa)	Transmittance (%)	Ref.
Beechwood	T: 0.1–0.7	NaClO_2_	PMMA	NW: 220 DL: 75 TW: 150	NW: 1.52 DL: 2.5 TW: 2.1	>10–70	[2]
Douglas fir (*Pseudotsuga menziesii*)	320 × 170 T: 0.6–2	NaClO_2_	Epoxy resin	NW_⊥_: 6.24 NW_//_: N/A TW_⊥_: 21.6 TW_//_: 92	N/A	>80	[10]
Balsa wood (*Ochroma pyramidale*)	20 × 20 T: 0.7–3.7	NaClO_2_	PMMA	NW: N/A DL: 10 TW: 95	NW: N/A DL: 0.22 T: 2.05	40–90	[1]
Pine, birch, and ash wood veneer	100 × 100 T: 1.5	H_2_O_2_, NaClO_2_	PMMA	100.7	N/A	80	[16]
Basswood	50 × 50 T: 3	NaOH; Na_2_SiO_3_, H_2_O_2_	Epoxy resin	NW_⊥_: <5 NW_//_: <45 TW_⊥_: <23.4 TW_//_: 45.4	NW_⊥_: N/A NW_//_: N/A T_⊥_: 1.22 T_//_: 2.37	80–90	[21]
Balsa wood	20 × 40 T: 0.8	NaClO	Polyvinyl alcohol (PVA)	NW_⊥_: 1.15 NW_//_: 18.8 TW_⊥_: 67 TW_//_: 143	N/A	90	[10]
Poplar wood (*Populus deltoides*)	20 × 20 T: 1	NaOH, Na_2_EDTA, MgSO_4_, and H_2_O_2_	PVA and PG	NW: 61.5 LMW: 33.3 T-PG0: 39.9 T-PG50: 22.6 T-PG100: 13.3	NW: 1.98 LMW: 1.49 T-PG0: 1.51 T-PG50: 0.80 T-PG100: 0.26	65–80	[28]
Basswood	T: ∼0.7	NaClO, NaClO_2_	Epoxy resin	NW_⊥_: 8.5 NW_//_: 55 TW_⊥_: 33.3 TW_//_: 44.4	N/A	90	[29]
Poplar wood (*P. adenopoda Maxim*)	25 × 25 and 50 × 50 T: 1	NaClO_2_	ATO/PMMA	NW: 78.3 DL: 21.9: T: 93.0 ATO_0.3–0.7_T: 96.4–113.1	NW: 3.74 DL: 1.71 T: 3.38 ATO_0.3–0.7_ T: 4.27–4.46	45–80	[7]
Silver birch wood (*Betula pendula*)	20 × 20; 20 × 20 T: 0.5–1.5	NaClO_2_	PEG/PMMA	NW: 129.6 TW-TES: 70.5	NW:14.5 T: 14.9	60–80	[19]
Balsa wood (*Ochroma pyramidale*)	20 × 20 T: 1, 1.5, 2, and 5	NaClO_2_	Epoxy resin	NW_⊥_: 1.02 NW_//_: 12.01 TW_⊥_: 4.12–63.05 TW_//_: 45.12–75.12	N/A	10–80	[15]
Basswood (*Tilia*)	20 × 20 T: 0.42	NaClO_2_	PMMA	NW: 121.9 DL: 84–8-114.3 TW: 152.4–171.4	N/A	10–60	[17]
Fir	100 × 100 100 × 200 T: 0.1	NaClO_2_	Cellulose nanofiber, Chitosan	NW_⊥_: 1 NW_//_: 75 TW_⊥_: 26 TW_//_: 258 TW_⊥_: 26 TW_//_: 171	15 13	75–80	[14]

## Data Availability

No new data were created or analyzed in this study. Data sharing is not applicable to this article.
